# Elucidating Sensitivity and Stability Relationship of Gold–Carbon Hybrid LSPR Sensors Using Principal Component Analysis

**DOI:** 10.1021/acsomega.2c03326

**Published:** 2022-07-27

**Authors:** Nikhil Bhalla, Preetam Kumar Sharma, Supriya Chakrabarti

**Affiliations:** † Nanotechnology and Integrated Bioengineering Centre (NIBEC), School of Engineering, Ulster University, Shore Road, BT37 0QB Jordanstown, Northern Ireland, United Kingdom; ‡ Heathcare Technology Hub, Ulster University, BT37 0QB Jordanstown, Northern Ireland, United Kingdom; § Department of Chemical Engineering, Loughborough University, Loughborough LE11 3TU, United Kingdom

## Abstract

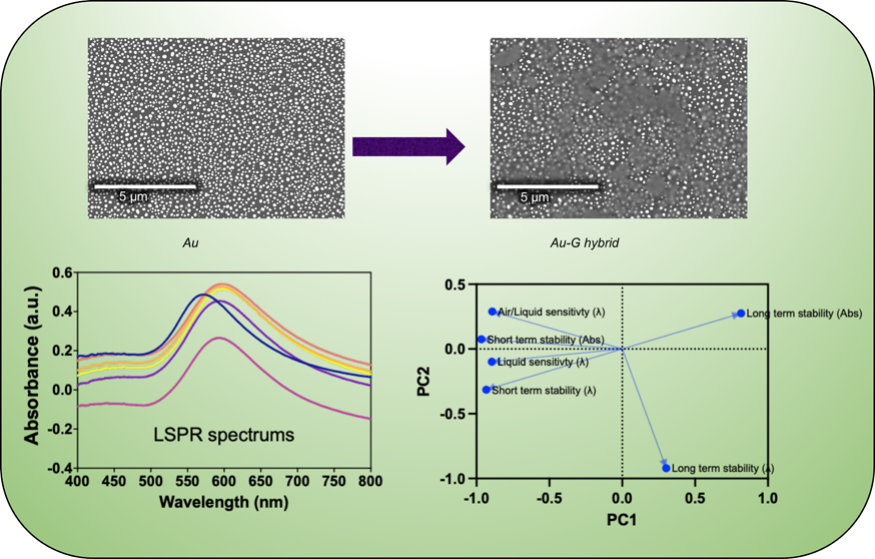

Sensitive localized surface plasmon resonance (LSPR) sensing is achieved using nanostructured geometries of noble metals which typically have dimensions less than 100 nm. Among the plethora of geometries and materials, the spherical geometries of gold (Au) are widely used to develop sensitive bio/chemical sensors due to ease of manufacturing and biofunctionlization. One major limitation of spherical-shaped geometries of Au, used for LSPR sensing, is their low refractive index (RI) sensitivity which is commonly addressed by adding another material to the Au nanostructures. However, the process of addition of new material on Au nanostructures, while retaining the LSPR of Au, often comes with a trade-off which is associated with the instability of the developed composite, especially in harsh chemical environments. Addressing this challenge, we develop a Au-graphene-layered hybrid (Au-G) with high stability (studied up to 2 weeks here) and enhanced RI sensitivity (a maximum of 180.1 nm/RIU) for generic LSPR sensing applications using spherical Au nanostructures in harsh chemical environments, involving organic solvents. Additionally, by virtue of principal component analysis, we correlate stability and sensitivity of the developed system. The relationship suggests that the shelf life of the material is proportional to its sensitivity, while the stability of the sensor during the measurement in liquid environment decreases when the sensitivity of the material is increased. Though we uncover this relationship for the LSPR sensor, it remains evasive to explore similar relationships within other optical and electrochemical transduction techniques. Therefore, our work serves as a benchmark report in understanding/establishing new correlations between sensing parameters.

## 1 Introduction

Localized surface plasmon resonance (LSPR) based spectroscopy, associated with noble metals, is a powerful transduction method used in a wide range of bio/chemical sensing applications.^1^ Compared to electrochemical^2^ or other contemporary optical transduction methods,^3^ LSPR offers several advantages such as less susceptibility to ambient noise or from the bulk media.^4^ This is due to the fact that LSPR has an evanescent delay length of around 20 nm which is 40–50 times less than the typical decay length in surface plasmon resonance (SPR) associated with planar films.^4,5^ For routine sensing using LSPR technique, usually a large area substrate consisting of metallic nanostructures such as gold (Au), silver (Ag), and aluminum (Al) is required.^6−8^ Most common supporting substrate chosen to create these nanostructures is either borosilicate glass or silicon-based substrates due to ease of metal deposition in terms of fabrication and cost associated with the materials.^9−11^ These substrates allow LSPR from the substrates to be measured in four optical modes, transmission (T-mode), reflection (R-mode), total internal reflection, and dark field scattering.^1^ The simplest of all set ups is the T-mode set up, also used in this study, where a light source and the detector is placed at two opposite sides of the substrates.^12^

The T-mode setup requires the substrates to be optically transparent and most commonly borosilcate glass, as also mentioned earlier, is used as a cost-effective transparent substrate for developing large area LSPR substrates.^13,14^ Furthermore, Au is the most common noble metal used for LSPR which has been developed in various geometries, such as nanospheres,^15^ nanorods,^16^ nanomushrooms,^17^ nanospikes,^18^ and many others.^19^ The different shapes, relative to each other, assist either in increasing the LSPR sensitivity toward a given target or to provide improved morphological features such as better stability/adhesion/survival of biomolecules/cells^20^ for enhanced LSPR bio/chemical sensing. Among these nanoscale Au geometries, the spherical-shaped nanostructures are one of the easiest to develop in terms of cost, time of fabrication, synthesis process, and in large area formats on borosilicate glass using the dewetting process.^21,22^ Additionally, the spherical shapes allow for optimal surface modifications on the Au nanostructures for biofunctionalization, as evident from a long history of conjugation with antibodies with spherical Au nanoparticles.^23−25^ The spherical-shaped Au nanoparticles are also demonstrated to have better survival rates for the long-term cell-based LSPR assays.^20^ Despite, these advantages, there is a limitation on the sensitivity of the spherical-shaped Au nanostructures which is either enhanced by change of the Au supporting substrate as shown in our previous work,^26^ in the case of the T-mode or by the addition of materials which will allow for enhancing the optical absorption of the Au.^27,28^ While sensitivity enhancement can be achieved using the addition of a new material, there is a trade-off in the form of short-term stability (stability of the measurement) and long-term stability (shelf life) of the Au-based LSPR.^29−31^ This is due to the fact that Au does not have oxidation as compared to materials used to enhance its LSPR-based refractive index sensitivity.^32^ This issue of stability is further amplified when the LSPR sensor requires operation in harsh chemical environments.^33^

Within this context, we have used graphene and graphene oxide, as an additive material on Au nanostructures to enhance the refractive index sensitivity of Au without affecting the short-term and long-term stability of the developed LSPR sensor in common solvents like methanol, ethanol, acetone, and isopropanol environments. Graphene is a two-dimensional layer of carbon atoms arranged in a honeycomb lattice, which has been theoretically investigated and experimentally demonstrated for exciting and propagating surface plasmons.^34^ It is indispensable to mention that due to its unique optoelectronic properties, graphene has found a wide range of applications including plasmonic sensors.^35^ However, sensing features of the graphene-AuNP hybrid include ultrahigh sensitivity, stability, and the affinity of biomolecule interactions that influence the detection of a diverse range of biomolecules with high specificity. These features imply that these composites have a promising role in future applications and the potential to be the preferred route of disease detection in clinical diagnosis applications.

## 2 Results and Discussion

**Figure 1:**
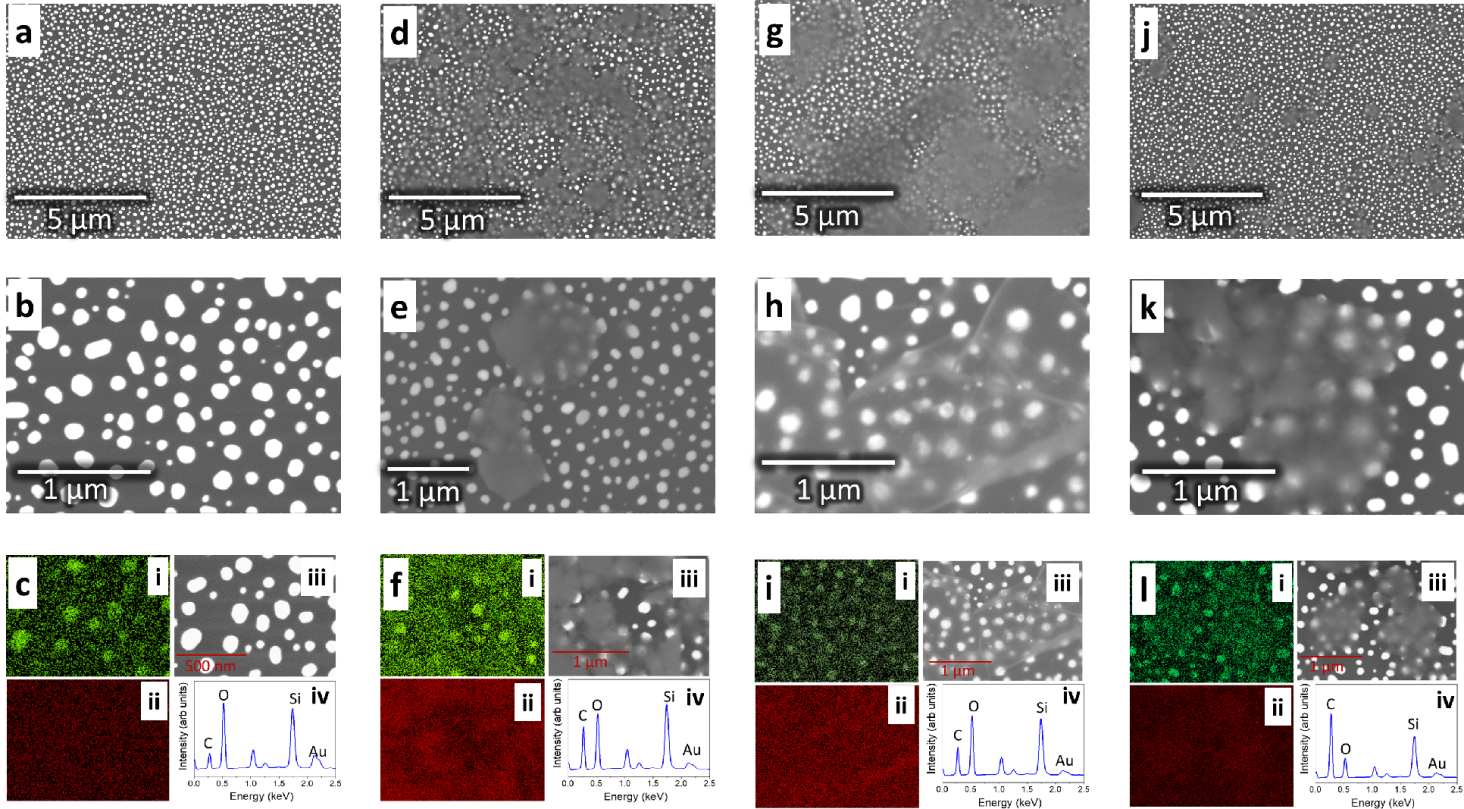
SEM and EDX: scanning electron microscopy images for the Au chip (a,b), G1 (d,e), G2 (g,h), and G3 (j,k). EDX analysis for the Au, G1, G2, and G3 samples is shown in figure (c), (f), (i), and (l), respectively. The subfigures in c, f, i, and l correspond to the (i) Au EDX map, (ii) C EDX map, (iii) SEM image, and (iv) EDX spectrum relating to the map. Note: Au refers to the Au nanoisland substrate and G1, G2, and G3 conventions are used for the graphene-AuNP hybrid with an average of 10 layers of graphene, the graphene-AuNP hybrid with an average of 20 layers of graphene, and the graphene oxide-AuNP hybrid with an average of 10 layers of graphene oxide, respectively.

We first perform surface characterization of the developed graphene-AuNP hybrid. It should be noted that AuNP refers to the Au nanoisland on the substrate, and G1, G2, and G3 conventions are used for the graphene-AuNP hybrid with an average of 10 layers of graphene, the graphene-AuNP hybrid with an average of 20 layers of graphene, and the graphene oxide-AuNP hybrid with an average of 10 layers of graphene oxide, respectively. The scanning electron microscopy (SEM) images of Au, G1, G2, and G3 samples are shown in Figure 1.

As shown in Figure 1a, b, the gold islands on the borosilcate glass are well-separated with an average size of 76 nm for Au islands (size distribution histogram shown in Figure S1). The Energy dispersive X-ray (EDX) analysis of Au chips (Au nanoislands on glass slides) is depicted in Figure 1c. Specifically, Figure 1c-i and c-ii shows the Au and C elemental map corresponding to Figure 1c-iii. The EDX spectrum obtained from the region is shown in Figure 1c-iv. The spectrum has major peaks of Si and O, which correspond to the borosilicate glass slide. Apart from this, peaks relating to Au and C were observed. The source of the C peak is attributed to the surface contamination/impurities of glass slide. The SEM and EDX analysis of G1, G2, and G3 samples are shown in Figure 1d–l. Upon comparing Figure 1d, g, j, the difference in the flake sizes are cleary observed. The flakes in G1 are ∼1 μm in size, G2 flakes are around 5 μm, and G3 flakes are submicron in size. The difference between flake sizes can also be observed at higher SEM magnification in Figure 1e, h, k. EDX analysis of G1, G2, and G3 samples is shown in Figure 1f, i, l. As shown in these figures, the areas covered by carbon from graphene show a lower EDX signal as compared to the exposed areas. Additionally, as expected, the C map shows higher intensity in the areas covered by graphene layers (sub figure ii of each image). Furthermore, the EDX signal corresponding to the C element was enhanced as compared to the bare Au chip.

**Figure 2:**
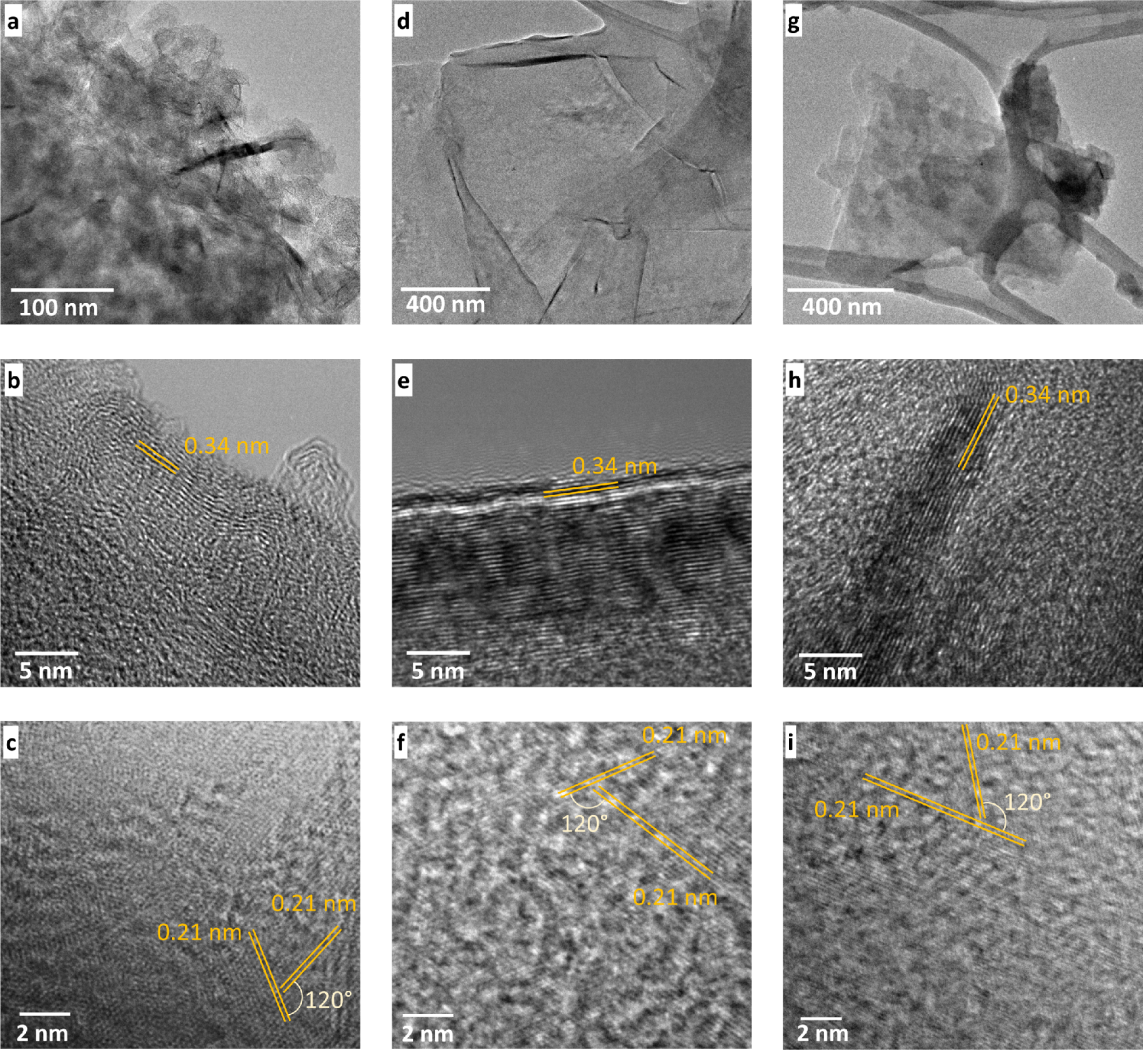
Transmission electron microscopy: low (top row) and high resolution (middle and bottom row) transmission electron images for (a–c) G1, (d–f) G2, and (g–i) G3 graphitic samples. Note: Au refers to Au nanoisland substrate, and G1, G2, and G3 conventions are used for the graphene-AuNP hybrid with an average of 10 layers of graphene, the graphene-AuNP hybrid with an average of 20 layers of graphene, and the graphene oxide-AuNP hybrid with an average of 10 layers of graphene oxide, respectively.

The transmission electron microscopy (TEM) images of the carbon from graphene and graphene oxide samples corresponding to G1, G2, and G3 are shown in Figure 2a–c, d–f, and g–i, respectively.

Upon comparing the images in the top row, the G2 sample showed regular and bigger flakes which are indicative of a higher order and a multilayer system. In comparison, G1 flakes are quite wrinkled and G3 flakes are smaller in size.This is because G3 represents the 4–10% edge-oxidized graphene oxide sample which makes G3 relatively weaker in terms of mechanical properties compared to graphene. For achieving uniform dispersion of graphene and graphene oxide in the NMP solution, the samples were sonicated for 1 h which caused the breaking of the flakes and make G3 flakes smaller in size compared to G1 and G2. To further determine the reason for the differences in the samples, higher resolution TEM analysis was performed. As shown in Figure 2b, e, and h, the spacings between graphitic carbon layers is 0.34 for all the samples, which corresponds to the 002 plane of graphitic carbon.^36^ The average number of layers, determined by analyzing multiple images (*n* ≥ 3) for each sample, are 9 ± 5 for G1, 18 ± 8 for G2, and 10 ± 3 for G3 sample. High-resolution TEM of the flakes also showed the distribution of atoms along the hexagonal plane. In all the planes, lattice spacing of 0.21 nm was measured, which corresponds to the 101 0 plane of the hexagonal lattice.^37^ As marked in the figures, planes at 120° inclination to the assigned planes with identical lattice spacings were also found, confirming the hexagonal symmetry. The hexagonal lattices of G2 and G3 (Figure 2f, i) have higher-ordered planes as compared to G1. This observation is supported by the low-resolution measurements where G2 and G3 flakes (Figure 2d, g) had better defined flakes as compared to G1, which is shown in Figure 2a.

Differences in the three graphitic samples were further investigated by determining their crystalline nature. From X-ray diffractograms (XRD) shown in Figure S2a, all three samples have diffraction peaks at 26.6, 42.7, and 54.6 degrees which correspond to 002, 200, and 004 planes of graphitic carbon.^38^ The XRD spectra indicates that G1 has low crystallinity compared to G2 and G3, which indicates the presence of more crystal structural defects in the G1 sample. Raman spectroscopy has also been utilized for the characterization of the graphitic carbon, as it can differentiate between graphite, graphene, and graphene oxide samples.^39^ As shown in Figure S2b, the samples have three major peaks, labeled as D, G, and 2D. The G peak corresponds to the graphitic characteristics of the material, D corresponds to the disordered graphitic material, and 2D is the 2nd harmonic of D. Therefore, the D/G ratio should provide indications toward the defect to the graphitization nature of the graphitic materials. The D/G ratio of the materials is in G1 > G3 > G2 order which matches well with electron microscopy and XRD analysis. We have also shared low-resolution SEM images of G1, G2, and G3 in Figure S3 to provide insights in our surface coverage of the adlayer on the AuNP.

After successful development of the graphene-AuNP hybrid, we extended our surface characterization studies to LSPR-based refractive index sensing.

**Figure 3:**
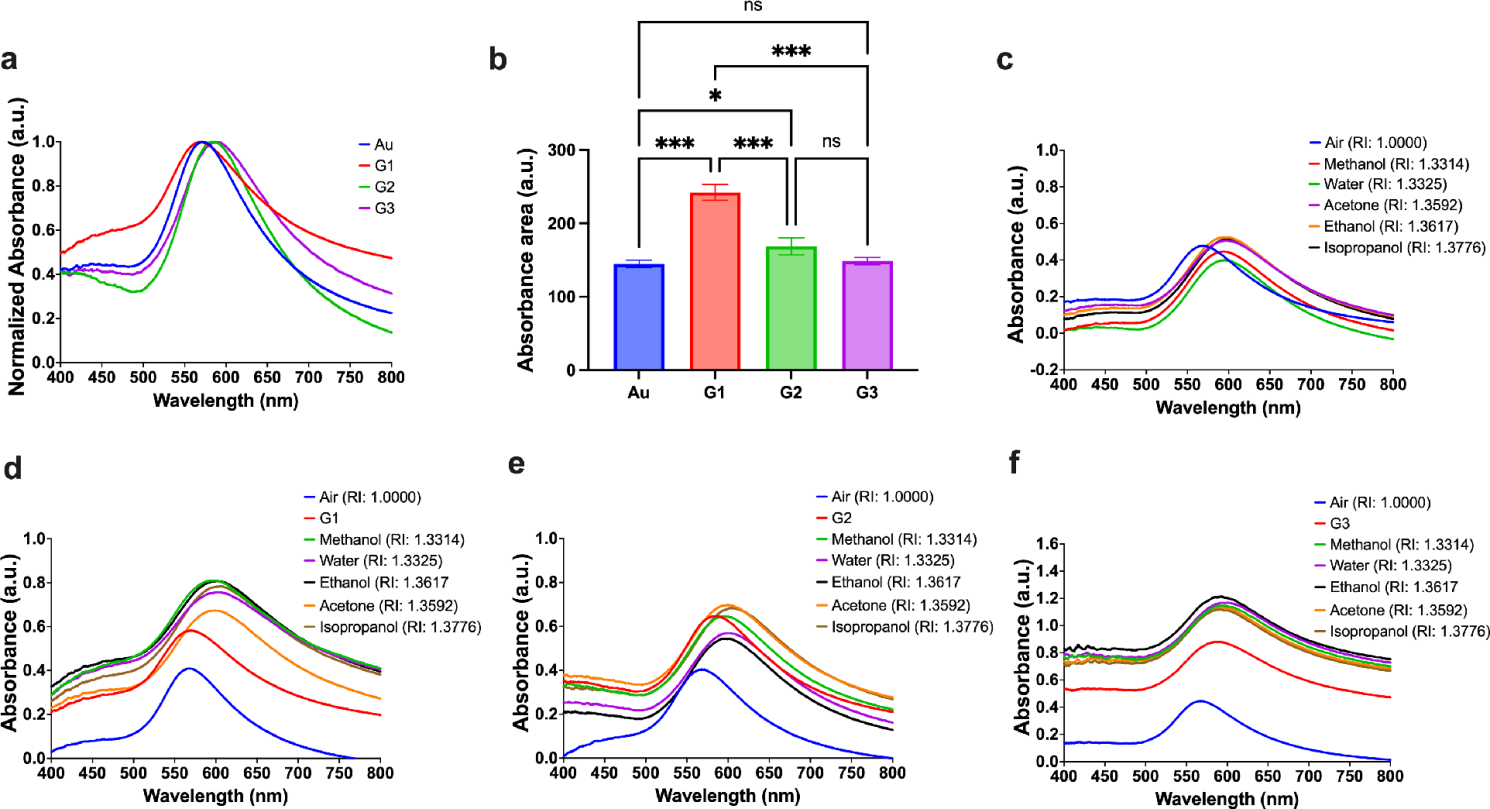
LSPR characterization: (a) Normalized absorbance vs wavelength of Au, G1, G2, and G3 showing LSPR peaks. (b) Tukey’s multiple comparisons test with alpha value = 0.05 performed on the absorbance change for Au, G1, G2, and G3. The number of stars indicate the degree of significance (the more the number of stars, the more significant the data). The LSPR responses of (c) Au, (d) G1, (e) G2, and (f) G3 are shown in various chemical environments. Note: Au refers to the Au nanoisland substrate and G1, G2, and G3 conventions are used for the graphene-AuNP hybrid with an average of 10 layers of graphene, the graphene-AuNP hybrid with an average of 20 layers of graphene, and the graphene oxide-AuNP hybrid with an average of 10 layers of graphene oxide, respectively.

Figure 3 shows LSPR characteristics of the developed graphene-AuNP hybrid. Essentially, Figure 3a shows normalized LSPR absorbance versus wavelength response of Au, G1–3, where a clear redshift in the wavelength and increase in total absorbance (area under the curve) is observed in the Au with addition of graphene/graphene oxide layers (G1–3). While detailed analysis of wavelength shifts is discussed in later sections, the curves in Figure 3a only depict typical response of the developed materials, and in Figure 3b we show a difference in the absorbance of the four materials. We observe statistically significant absorbance change in G1 and G2 compared to Au, suggesting that graphene layering on the Au leads to enhanced absorbance of light in comparison to the graphene oxide (see no significant statistical difference between G3 and Au). Note that this statistical analysis is performed using Tukey’s multiple comparisons test with alpha value = 0.05.

After the identification of the LSPR characteristics, we tested the developed materials for refractive index sensing in harsh chemical environments. We conducted this test by exposing the materials to the following solutions: water (RI: 1.3325) and harsh organic solvents: methanol (RI: 1.3314), acetone (RI: 1.3592), ethanol (RI: 1.3617), and isopropanol (RI: 1.3776). Changes in absorbance and wavelength were observed upon change in the local refractive index as shown in Figure 3c–f, characteristic plots showing refractive index changes for Au, G1, G2, and G3, respectively. We analyze these RI changes in detail for multiple sets of experiments (*n* ≥ 6) for all developed materials, seeFigure 4.

**Figure 4:**
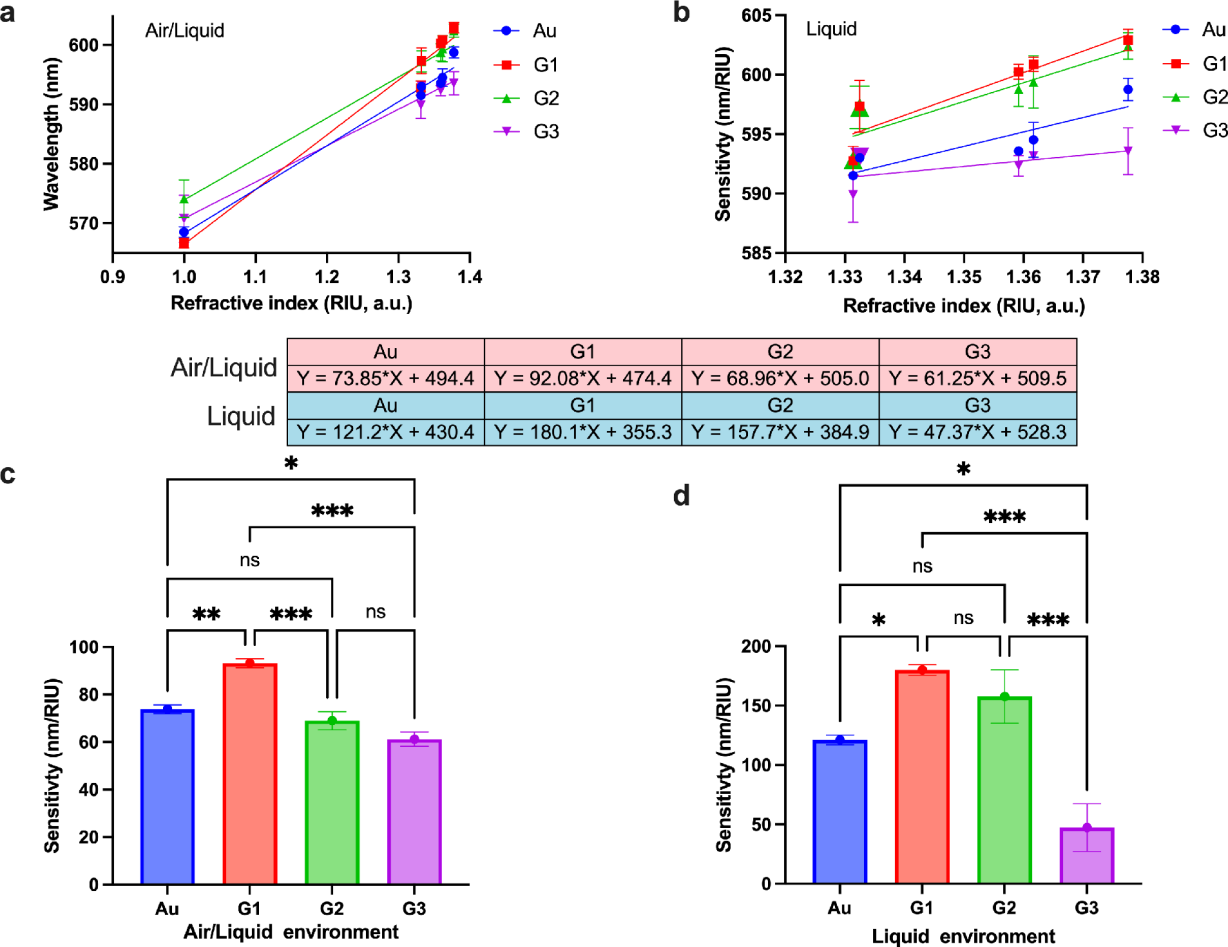
Refractive index sensitivity analysis: (a) refractive index vs wavelength plot for Au, G1, G2, and G3 in air/liquid environment. (b) Refractive index vs wavelength plot for Au, G1, G2, and G3 in a liquid only environment. Panels (c, d) show multiple comparison tests on Au, G1, G2, and G3 performed using Tukey’s test on refractive index sensitivities obtained for experiments conducted in the air/liquid environment and liquid-only environment, respectively. Note: Au refers to Au nanoisland substrate and G1, G2, and G3 conventions are used for the graphene-AuNP hybrid with an average of 10 layers of graphene, the graphene-AuNP hybrid with an average of 20 layers of graphene, and the graphene oxide-AuNP hybrid with an average of 10 layers of graphene oxide, respectively.

We plot the RI changes versus wavelength by (1) considering the native response of the respective substation in the air and liquid environment, Figure 4a, and by (2) exclusively considering the aforementioned sensor response in the liquid environment only, Figure 4b. In both plots, we fit the data with a linear regression line where its goodness of fit is between 0.95 and 1 (*R*^2^ value). The slope of this linear regression provides refractive index sensitivity of the material in nanometer per refractive index units (nm/RIU). The slopes are summarized in the table embedded inside Figure 4. Among the developed hybrids, the highest RI sensitivity is achieved by G1 followed by G2 and G3 in air/liquid environments. This suggests that pristine graphene layers (G1 and G2) are better suited to combine with Au as compared to its combination with graphene oxide (G3) for LSPR-based refractive index sensing in air/liquid environments. Additionally, G1 also enhanced the overall sensitivity of Au from 73.85 nm/RIU to 92.06 nm/RIU. Furthermore, analyzing the behavior of the sensors exclusively in the liquid environments, all graphene-modified Au substrates, G1 = 180.1 nm/RIU and G2 = 157.7 nm/RIU, yielded higher sensitivities than the Au = 121.2 nm/RIU. However, the graphene oxide modified substrate, G3, achieved a reduced sensitivity of 47.37 nm/RIU.

We also performed Tukey’s multiple comparison test on the obtained sensitivity values for both air/liquid, seeFigure 4c, and liquid only conditions, see Figure 4d. The analysis revealed significant differences between RI sensitivity of G1 and rest of the substrates in air/liquid environments. Additionally, in liquid only conditions, G1 provides the significantly higher sensitivity when compared to Au and graphene oxide modified G3 substrates. It should be noted that in the liquid-only condition, G1 and G2 do not have significant differences in their sensitivities as compared to their differences in the air/liquid environments; however, G1 has a higher mean sensitivity than G2 in both conditions. Nevertheless, it provides another evidence (in addition to the aforementioned statistical analysis) that graphene modification is better than graphene oxide modification on spherical Au nanostructures for LSPR sensing applications.

**Figure 5:**
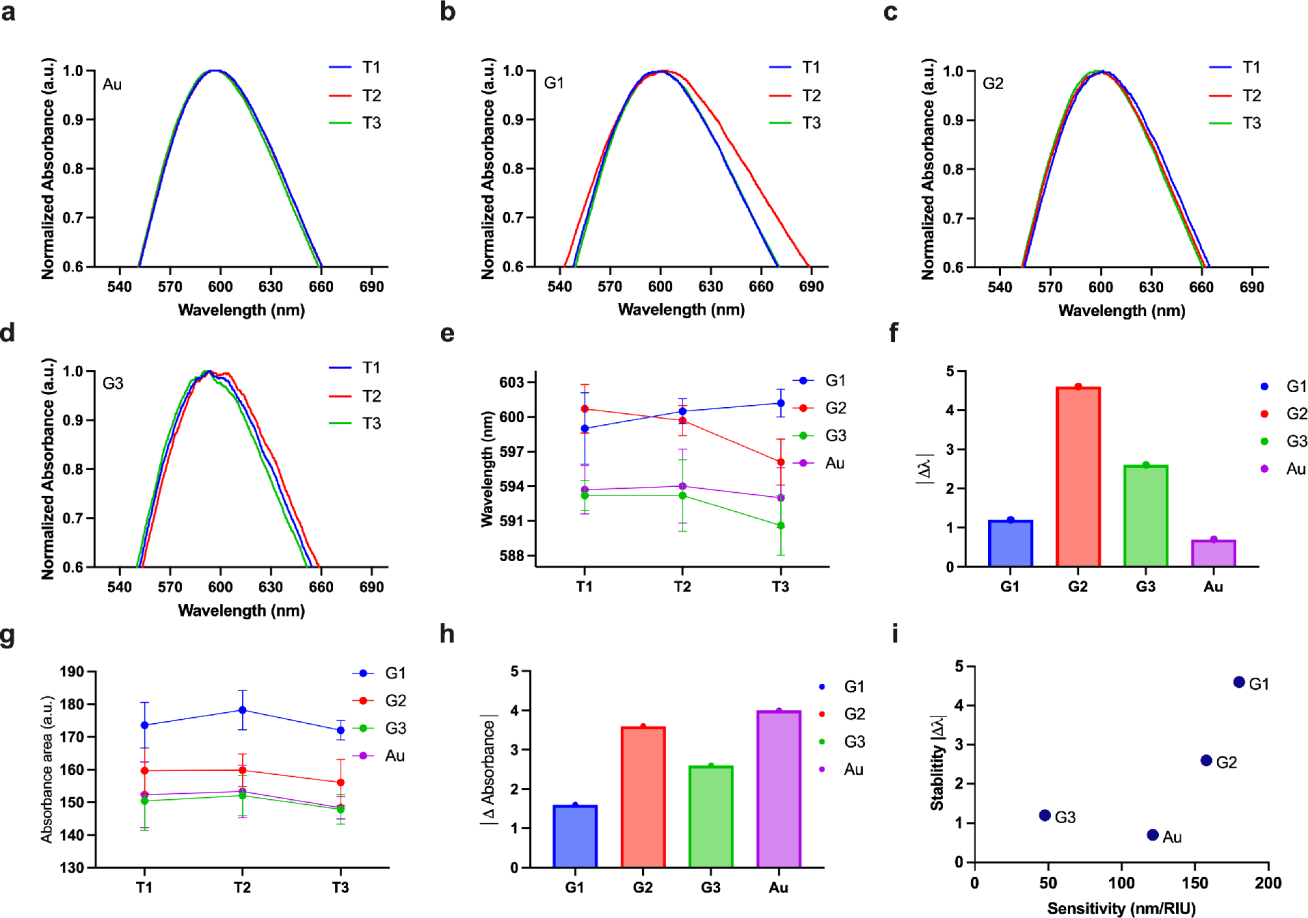
Short-term stability analysis. (a–d) Normalized absorbance vs wavelength plot for Au, G1, G2, and G3, respectively, at time periods T1, T2, and T3. (e) Wavelength changes at time T1, T2, and T3 for Au, G1, G2, and G3. (f) Wavelength change between T1 and T3 for Au, G1, G2, and G3. (g) Absorbance changes at time T1, T2, and T3 for Au, G1, G2, and G3. (h) Absorbance change between T1 and T3 for Au, G1, G2, and G3. (i) Sensitivity vs stability plot for Au, G1, G2, and G3. Note that T1 = 20 min, T2 = 40 min, and T3 = 60 min. Note: Au refers to Au nanoisland substrate, and G1, G2, and G3 conventions are used for the graphene-AuNP hybrid with an average of 10 layers of graphene, the graphene-AuNP hybrid with an average of 20 layers of graphene, and the graphene oxide-AuNP hybrid with an average of 10 layers of graphene oxide, respectively. The subfigures (a–d) are representative spectrum corresponding to the experimental conditions which yield shifts within the standard deviations shown in (e) and (g) subfigures.

One challenge associated with the modification of spherical Au nanostructures with another material, such as graphene or graphene oxide in our case, is to ensure the mechanical stability of the Au on its substrate and its associated LSPR properties. Within this context, we study short and long-term stability of G1, G2, and G3 and compare it with the stability of Au. The short stability is performed by exposing the substrates to acetone which we consider as the most harsh chemical in the set of organic solvents chosen in our study for refractive index characterization. The total exposure lasted for 60 min and at each 20 min interval (T1, T2, and T3) LSPR spectra were acquired, see Figure 5a, b, c, d for Au, G1, G2, and G3, respectively. The acquired LSPR spectrum was analyzed for changes in wavelength (Figure 5e, f) and absorbance (Figure 5g, h) as reported as mean values (*n* ≥ 3). Note Figure 5e, g represents the absolute changes in the wavelength and total absorbance, whereas Figure 5f, h shows shifts in the wavelength and absorbance between T1 and T3. From the wavelength shifts, it is clear that Au is more stable compared to all other modifications. However, the differences in the stability or changes in the wavelength are minuscule, and since no significant differences are found between any of the pairs (therefore not shown in the figure), we can confirm that all modifications do not hamper the LSPR stability of the pristine Au substrate. Additionally, from a qualitative perspective with respect to the wavelength and absorbance change, we can say that among the various modifications, the graphene oxide modified Au (G3) is more stable in comparison to the G1 and G2. This can also be attributed to the higher sensitivity of the G1 and G2 as compared to G3, see sensitivity versus stability plot in Figure 5i. Clearly we can observe that as sensitivity of the material (in our case Au) increases, the short-term stability of the material falls down. However, no quantitative assertion can be made on the trend of the data, and therefore, we do not fit any of the points with linear regression or some other compatible trendline fit.

**Figure 6:**
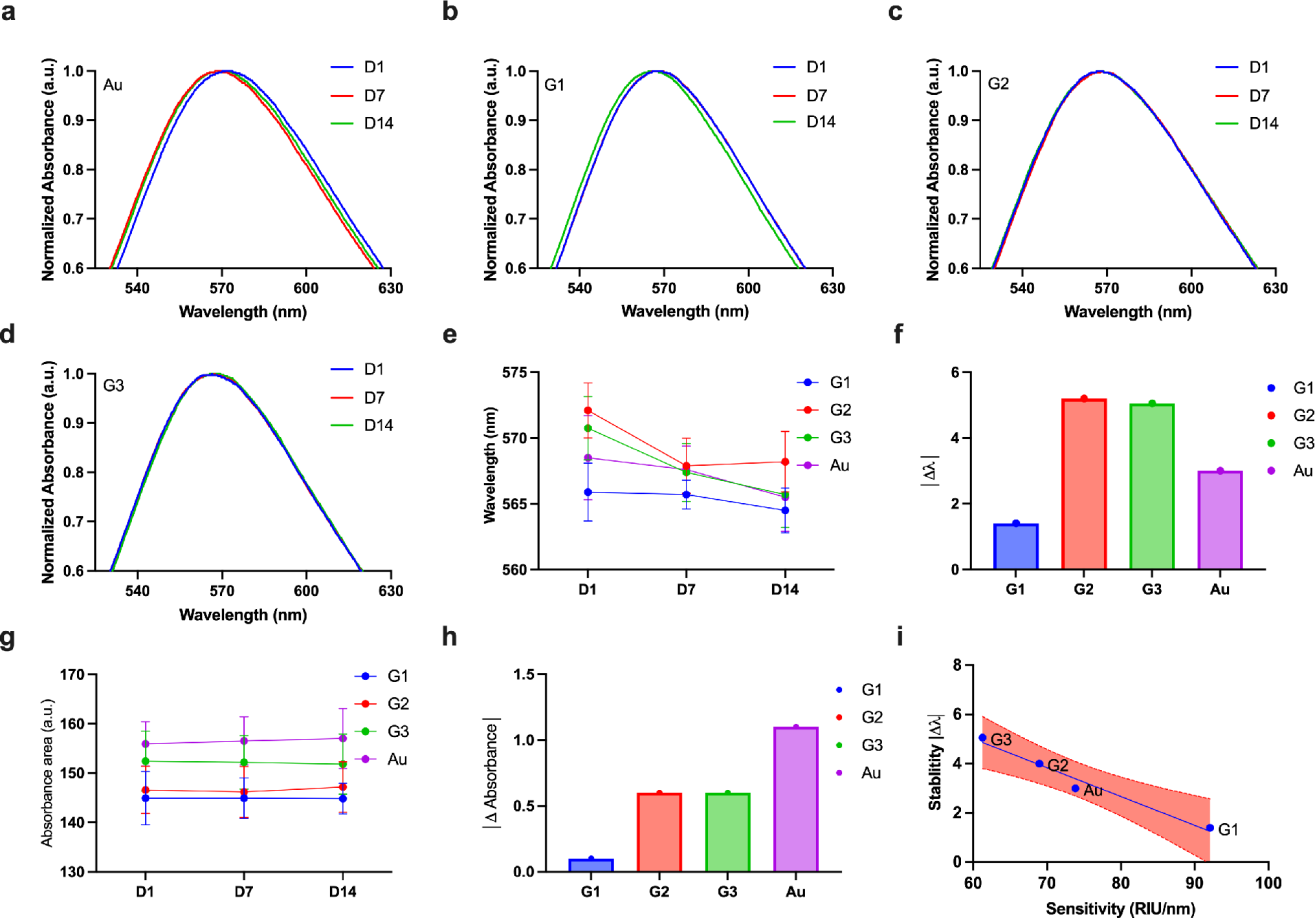
Long-term stability analysis: (a–d) Normalized absorbance vs wavelength plot for Au, G1, G2, and G3, respectively, on D1, D7, and D14. (e) Wavelength changes on D1, D7, and D14 for Au, G1, G2, and G3. (f) Wavelength change between D1 and D14 for Au, G1, G2, and G3. (g) Absorbance changes on D1, D7, and D14 for Au, G1, G2, and G3. (h) Absorbance change between D1 and D14 for Au, G1, G2, and G3. (i) Sensitivity vs stability plot for Au, G1, G2, and G3. Note that D1 is day 1, D7 is day 7, and D14 is day 14. Note: Au refers to the Au nanoisland substrate and G1, G2, and G3 conventions are used for the graphene-AuNP hybrid with an average of 10 layers of graphene, the graphene-AuNP hybrid with an average of 20 layers of graphene, and the graphene oxide-AuNP hybrid with an average of 10 layers of graphene oxide, respectively. The shaded area within (i) represents the confidence interval of 95% for the line fitted using linear regression with a *R*^2^value of 0.98. The subfigures (a–d) are representative spectrum corresponding to the experimental conditions which yield shifts within the standard deviations shown in (e and g) subfigures.

We also analyzed the long-term stability of the developed material which can contribute to the shelf life of the material. This stability is measured by recording the wavelength of the developed material on day 1, day 7, and day 14 exclusively in the air environment (D1, D7, and D14), see Figure 6a, b, c, and d for Au, G1, G2, and G3, respectively. Similar to short-term stability analysis, the acquired LSPR spectrum was analyzed for changes in wavelength (Figure 6e, f) and absorbance (Figure 6g, h) by extracting mean values for the change (*n* ≥ 3). Here, Figure 6e, g represents the absolute changes in the wavelength and total absorbance, whereas Figure 6f, h shows shifts in the wavelength and absorbance between D1 and D14. From both wavelength and absorbance shifts, the most stable substrate is found to be G1 and a direct linear relationship between sensitivity and stability (in air/liquid environment as the long-term stability is measured in air) is observed, see Figure 6i. Note that the shaded portion of the linear fit represents the 95% confidence interval with a good of fit of 0.98 (*R*^2^ value of the fit). Therefore, we can conclude that the higher the overall sensitivity of the material (in air/liquid), the more stable it is in air. Even though the changes in the wavelength and absorbance in all substrates is not large, the higher absorbance and wavelength shifts in G2 and G3 can be attributed to the higher amount of oxidation of the graphene (in G2 as it has a high number of graphene layers) and graphene oxide (G3) layers.

**Figure 7:**
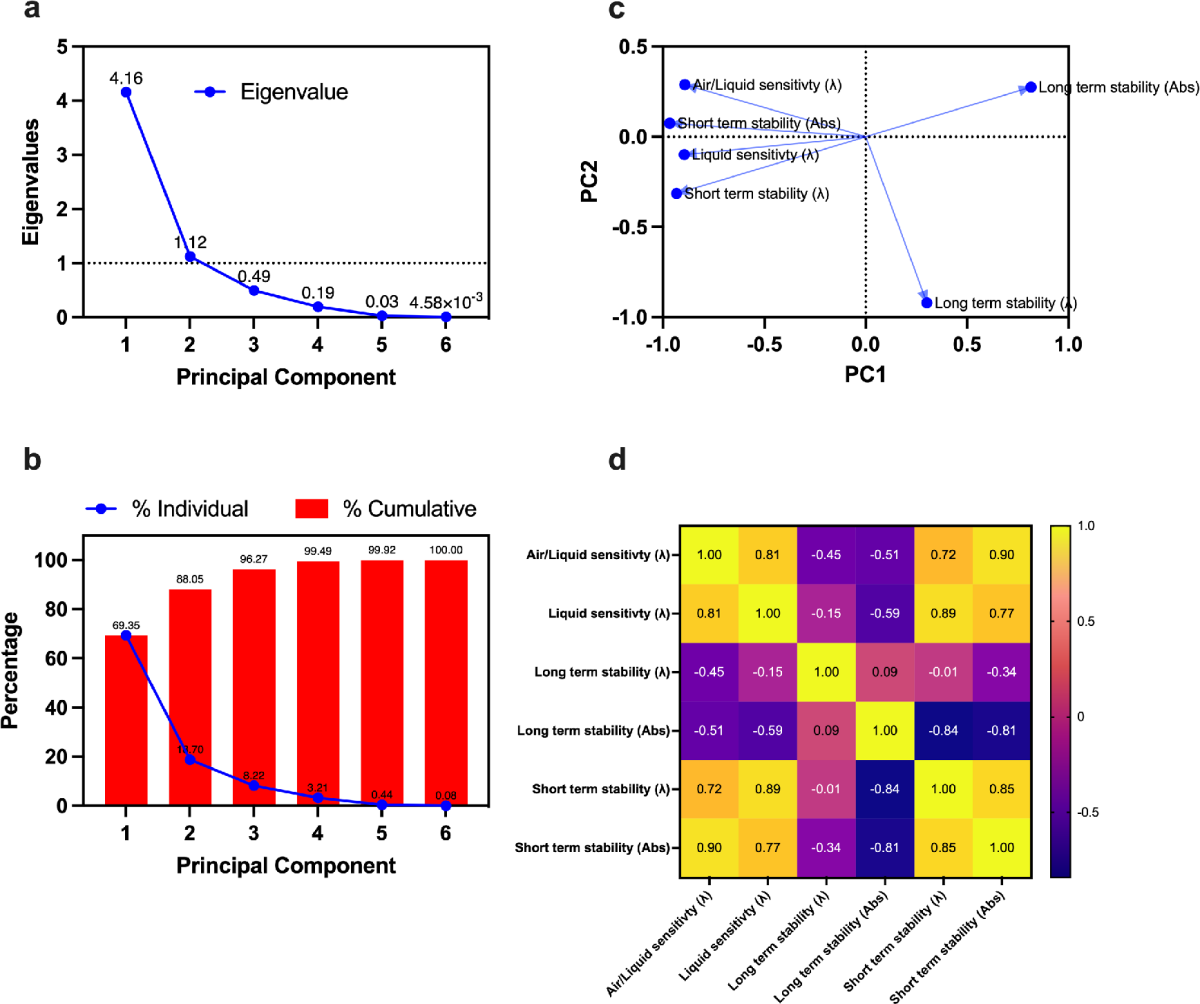
Stability vs sensitivity PCA. Panel (a) shows eigenvalues of all principal components. Panel (b) demonstrates the contribution of each principal component toward the variance in the given data. The principal components 1 and 2 account for more than 88.05% variance, and therefore, PC1 and PC2 are selected to show the loading plot in panel (c); Panel (d) shows a matrix showing Pearson correlation (*r*) between all variables used within PCA.

We also analyze the stability and sensitivity of all the developed sensors by the virtue of principal component analysis (PCA), see Figure 7. The aim of the PCA analysis was to elucidate a general trend between sensitivity and stability of the LSPR sensor. We performed PCA using the multiple variable analysis tool built within GraphPad Prism 9 software. The principal components are chosen based on the Kaiser-Guttman rule, which allows us to consider all principal components with eigenvalues greater than 1. From Figure 7a, it is evident that there are 2 principal components, PC1, and PC2, which have eigenvalues greater than 1, together accounting for 88.05% of the variance in the data, Figure 7b, and are therefore used for further analysis. Note that all principal components are given by a linear combination of the input variables, where the weighting coefficients are called loading, which is plotted in Figure 7c. In the loading plot, points which lie closer to each other are closely related parameters. More specifically, if two points lie on a line, we can consider one point as completely redundant to describe the behavior of the analyzed system. Within this context, it can be observed that short-term stability, both wavelength absorbance shifts, in all developed samples are directly affected by changes in the sensor sensitivity (also see correlation matrix in Figure 7d), further endorsing that the sensitivity and short stability shown earlier in Figure 5e might be directly proportional to each other, even though linear regression cannot be used to fit the data trend. On the contrary the shelf life, which is associated with the long-term stability of the sensor is inversely related to the sensitivity of the sensor, see negative correlation of the long-term stability with other variables in Figure 7d. This can possibly be attributed to the fact that higher sensitivity of the pristine Au sample is usually achieved by additive materials (as also shown in our work) which over a period of time can undergo degradation due to oxidative effects from the ambient environment which may contribute toward its decrease in shelf life.

## 3 Conclusions

Graphene with Au is better suited for LSPR sensing application as compared to the combination of Au with graphene oxide. There exists an apparent linear relationship between stability and sensitivity of the LSPR substrates. The relationship suggests that the shelf life of the material is proportional to its sensitivity, while the stability of the sensor during the measurement in the liquid environment decreases when the sensitivity of the material is increased. Though the developed sensor in this work is used for refractive index sensing, we can extend the use of these sensors to a wide range of bio/chemical sensing involving cells, proteins, and nucleic acid based entities. For example, in recent times we have developed several LSPR materials and sensors,^40−42^ which require improvement in their sensing performance from the perspective of sensor stability and sensitivity. Therefore, the developed LSPR substrate serves as a generic sensing platform with potential use in bioassay applications.

## 4 Experimental Section

### 4.1 Materials

Graphene with different number of layers and graphene oxide materials in the powder form were directly purchased from Sigma-Aldrich, UK. The sample G1 is graphene consisting of short stacks of graphene sheets having a platelet shape. The typical thickness is a few nanometers and consists of 10 to 12 graphene layers. The sample G2 is the graphene sample which has up to 20 layers of graphene. The sample G3 is graphene oxide powder, which consists of an average of 10 to 12 layers and contains functional organic groups. The oxygen content in G3 is less than 5%; the majority groups are hydroxyls (OH). For making the Au-graphene-layered hybrid, the graphene powder samples were first mixed with *N*-methyl-2-pyrrolidone (NMP) solution under magnetic stirring for 10 min, and a concentration of 4 mg/mL was maintained. Then the solution was further sonicated for 1 h to achieve uniform dispersion of the graphene in the NMP solvent. Finally, the graphene dispersion was drop-casted on Au-coated borosilicate substrate incubated for 15 min and then heated on a hot plate at 100 °C for allowing the solvent to evaporate. The Au nanoparticle chip was purchased from NanoSPR, USA.

### 4.2 LSPR Measurements

The instrument used to study the LSPR response is a custom-assembled benchtop tool designed by combining discrete optical components necessary for sample illumination and light collection. Briefly, the assembly involves two fiber optics patch cords, one connected with a halogen light source (DH 2000-S-DUV) and the other connected to a spectroscope (FLAME T-XR1-ES), which were all purchased from Ocean Insight, UK. The fiber optics was aligned for light exposure and collection of light in the transmission setup using the RTL-T stage purchased from Ocean Insight, UK. Before taking any signal from the spectroscope, the system was calibrated for background and maximum light absorbance using dark and light spectrum modes. The LSPR signal was then recorded in an absorption mode by observing the wavelength dependence of the light absorbed by nanocomposite via the OceanART software (cross-platform spectroscopy operating software from Ocean Insight). The data analysis is performed using GraphPad Prism software. For all experiments, at least 6 chips were utilized for the measurement of each type of substrate modification. One light spot, covering a 3 mm circular area, covers whole chip and ensures that the measurement area is not changed from measurement to measurement. See the setup in our past papers where we were able to control the size of the spot without the need to measure multiple spots.^43,44^ Here, the measurement probe fits exactly on the top of our LSPR sensor surface.

### 4.3 Materials Characterization

Transmission electron microscopy (TEM) measurements at various magnifications were performed at 200 kV using the JEOL JEM 2100F instrument. The samples for TEM analysis were prepared by depositing dispersions in NMP on the TEM grid followed by drying under an IR lamp for 4 h. Similarly, the samples for scanning electron microscopy (SEM) measurements were prepared by depositing the dispersions on a Au chip. The SEM measurements were performed using a Hitachi SU5000 instrument at 10 kV bias. Due to the nonconductive nature of the Au islands chip, the measurements were performed at low vacuum using a BSE detector. The energy dispersive X-ray analysis was performed using an Oxford Instruments X-Max^N^ detector connected to the SEM at 10 kV and low vacuum. X-ray diffraction measurements on the deposited thin films were performed using a Panalytical Empyrean Series 3 system in the 10–80° range at a grazing incidence of 2°. Renishaw in Via Qontor Raman spectrometer irradiated with 514 nm laser at 20× magnification was utilized for obtaining Raman spectra.

## Supporting Information Available

Additional information describing Tukey’s multiple comparison test, morphological size distribution of AuNP on glass, XRD, Raman, and the figure of merit for the LSPR sensor used in the work (PDF)
