# Supplementary material for: Elucidating Sensitivity and Stability Relationship of Gold–Carbon Hybrid LSPR Sensors Using Principal Component Analysis
Source: ACS Omega. 2022 Jul 27;7(31):27664–73. doi: 10.1021/acsomega.2c03326 (PMC9366941; doi:10.1021/acsomega.2c03326)
Supplement: Supplementary file 1 — ao2c03326_si_001.pdf [file ao2c03326_si_001.pdf]

Elucidating sensitivity and stability relationship of gold-carbon hybrid LSPR sensors using principal component analysis

Nikhil Bhalla[a,b,*], Preetam Kumar Sharma[c,*], Supriya Chakrabarti[a,*]

[a]*Nanotechnology and Integrated Bioengineering Centre (NIBEC), School of Engineering, Ulster University, Shore Road, BT37 0QB Jordanstown, Northern Ireland, United Kingdom*

[b]*Heathcare Technology Hub, Ulster University, BT37 0QB Jordanstown, Northern Ireland, United Kingdom*

[c]*Department of Chemical Engineering, Loughborough University, 0Loughborough LE11 3TU, United Kingdom*

**Corresponding authors: n.bhalla@ulster.ac.uk; P.Sharma@lboro.ac.uk s.chakrabarti@ulster.ac.uk*

Tukey's multiple comparison test

Tukey test compares every mean (of different groups in a given dataset) with every other mean. We have used in-built tool within the software GraphPad Prism to perform the Tukey's test, which allows comparison of unequal sample sizes. This test take into account the scatter of all the groups. This gives a more precise value for scatter (Mean Square of Residuals) which is reflected in more degrees of freedom.

When we compare mean of one group to the means of other group, the test compares the difference between means to the amount of scatter, quantified using information from all the groups, not just groups under comparison. This gives the test more power to detect differences. The results are a set of decisions: "statistically significant" or "not statistically significant". As aforementioned, these decisions take into account multiple comparisons. The test also compute a confidence interval for the difference between the two means. Note that this confidence interval accounts for multiple comparisons. In our tests, we have chosen 95% intervals, and therefore we are 95% confident that all of the intervals contain the true population value.

Morphological size distribution of gold nanoislands on glass

Figure S1 below shows the size distribution of Au NP islands on borosilicate glass substrate, which indicates a range of few nm to 200 nm with an average of 76 nm island size. Majority of the island size lies within 100 nm.

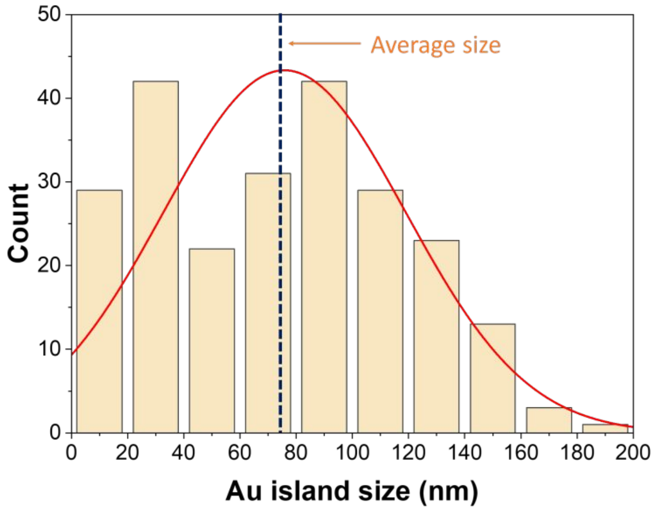

Figure S1. Size distributions of Au islands on glass substrate

XRD and Raman characterisation of G1-G3 on gold nanoislands

Figure S2a shows the comparative XRD spectrum of G1, G2 and G3 samples. The G1 has the lower crystallinity, whereas G2 and G3 shows good crystallinity which infers that G1 sample has more defects and random orientation of crystal lattice. Figure S2b shows the Raman spectra of G1, G2 and G3 samples and a decreasing trend of D/G (defect to graphitization) ration can be seen from G1 to G2 to G3. This also indicates the presence of more defects in G1 sample.

G1, G2 and G3 conventions are used for graphene-AuNP hybrid with an average 10 layers of graphene, graphene-AuNP hybrid with an average 20 layers of graphene and graphene oxide-AuNP hybrid with an average 10 layers of graphene oxide respectively.

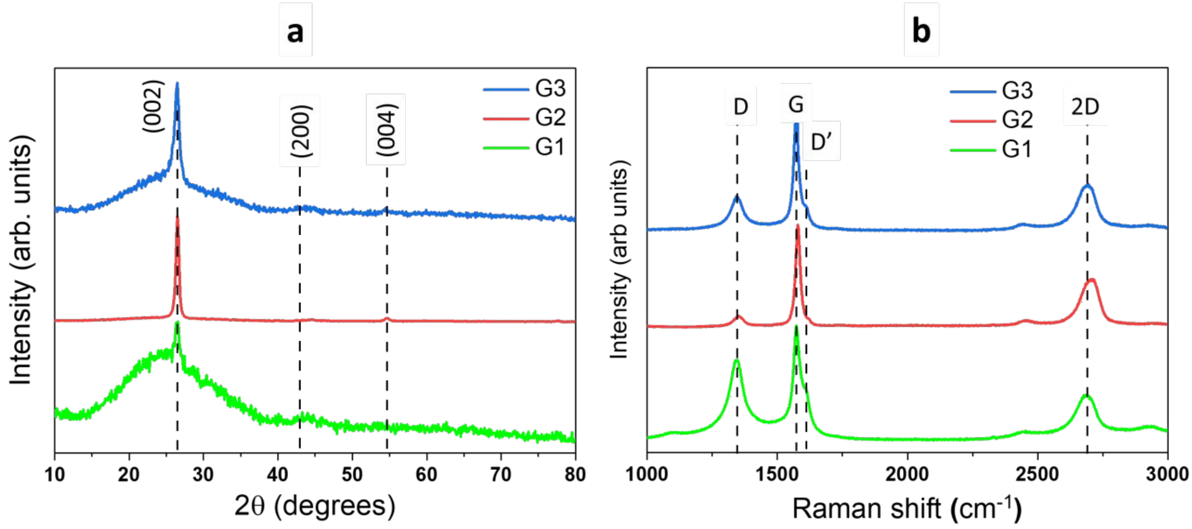

Figure S2. (a) XRD and (b) Raman spectra of G1-G3 samples.

Low resolution SEM imaging of G1,G2 and G3

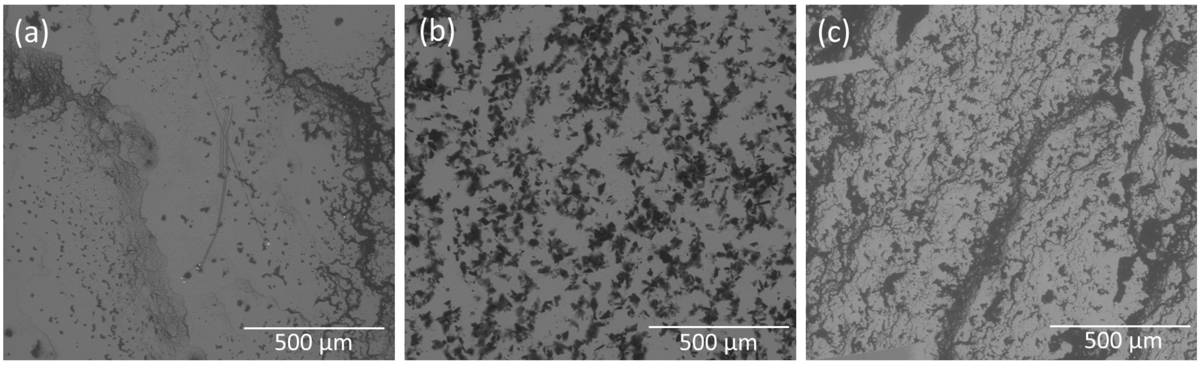

Figure S3. Low resolution SEM images of (a) G1-Au, (b) G2-Au and (c) G3-Au samples.

Figure of merit calculation for Au, G1 , G2 and G3

The figure of merit (FOM) of LSPR sensors is computed by calculation the full width and half maximum (FWHM) of the LSPR spectrum and then dividing it by the sensitivity obtained for a given substrate. The table below shows the sensitivity, FWHM and FOM of the develop sensors. Please note that the FOM is calculated by consideration of mean FWHM and mean sensitivity values only. Here FWHM is considered for all samples tested with 5 different organic solvents

Table S1. Table S1. LSPR sensor FOM in air/liquid environment

	FWHM	Sensitivity	FOM
Au	81.23 ± 4.25	73.85 ± 3.27	0.91
G1	83.13 ± 3.21	92.08 ± 4.72	1.11
G2	80.64 ± 6.17	68.96 ± 6.53	0.86
G3	84.64 ± 3.83	61.20 ± 7.34	0.72

Table S2. LSPR sensor FOM in liquid environment

	FWHM	Sensitivity	FOM
Au	84.64 ± 3.91	121.20 ± 3.12	1.43
G1	85.79 ± 2.64	180.10 ± 7.53	2.10
G2	86.42 ± 4.22	157.70 ± 39.01	1.82
G3	85.13 ± 1.31	47.35 ± 34.70	0.55
